# Enhanced Antibacterial Activity of Hydrophobic Modified Lysozyme Against Gram-Negative Bacteria Without Accumulated Resistance

**DOI:** 10.3390/molecules30020232

**Published:** 2025-01-09

**Authors:** Zhenhui Li, Song Lin, Mei Zhu, Xiaoman Liu, Xin Huang

**Affiliations:** School of Chemistry and Chemical Engineering, Harbin Institute of Technology, Harbin 150001, China; lizhenhui1993@163.com (Z.L.); 18204623951@163.com (S.L.); zhumeipolymer@163.com (M.Z.); liuxiaoman@hit.edu.cn (X.L.)

**Keywords:** antibacterial, lysozyme, hydrophobic modification, biocompatibility

## Abstract

Macromolecule bactericides present challenges such as low biocompatibility and not being biodegradable, so broad-spectrum bactericides without accumulated bacteria resistance are now in urgent demand all over the world. Lysozyme, a kind of wide-spread natural enzyme easily extracted from nature, has become attractive for agriculture and medicine use. However, Gram-negative bacterial strains are highly resistant to natural lysozymes, which limits their practical application. In this study, rather than directly modifying antibacterial-active substance with lysozyme, we show an effective way to improve antibacterial performance by altering the hydrophobic functional groups of natural lysozymes and synthesize a type of hydrophobic modified lysozyme (HML). Compared with other modification methods, the antibacterial performance has been increased by over 50%. We investigated its antibacterial mechanism against Gram-negative bacteria and showed that HML could be used to treat pathogenic bacteria without obvious accumulated resistance appearance, which is a great advantage over commercial antibiotics. Overall, it is anticipated that HML could be potentially applied to food safety, infection therapy, and enzyme-medicine applications.

## 1. Introduction

The abuse of antibiotics in agriculture and clinical medicine use [1,2] has introduced the problem that natural bacterial strains quickly accumulate resistance against commercial bactericides through the screening effect of antibiotics. Furthermore, multi-drug resistant (MDR) bacteria have appeared, such as methicillin-resistant Staphylococcus aureus (MRSA) and serial strains of Gram-negative bacteria, which have become a huge threat to global health security [3,4]. Therefore, there is an urgent need for broad-spectrum bactericides that not only exhibit high antibacterial activities against pathogenic bacteria, but also do not exhibit the behavior of accumulating bacterial resistance against the synthesized bactericide. Aiming to solve this problem, researchers have devoted their efforts to inorganic nanoparticle bactericides [5,6], metal–organic framework bactericides [7,8], and polymer macromolecule bactericide [9,10,11], to obtain an ideal bactericide.

Polymer bactericide is one of the most feasible choices among all the research directions. The main feature of polymer bactericides is their high antibacterial performance against pathogenic bacterial strains and excellent physical/chemical stability in applications. As is known, the antibacterial performance could be adjusted by the side functional groups linked to the main chain. Yang et al. systematically explored the impact of hydrophobic subunits and chain length variation on the antibacterial and antibiofilm activity of β-peptide polymers, and found that the synthesized bactericide showed low toxicity to human periodontal ligament fibroblasts [12]. Meanwhile, the main chain structure of a polymer bactericide is usually composed of a highly stable alkane chain, which limits the ability of the main chain of the polymer bactericide to exhibit further functionalities, except for supporting the side functional groups. Although polymer bactericides benefit from their high molecular weight and stable chemical structure, the outstanding physical/chemical stability of the alkyl main chain makes it difficult for bacteria to be normally degraded by human metabolism, limiting their application in clinical and agricultural use. Previous research has demonstrated that the antibacterial properties of polymer bactericides are usually reflected by their free-side functional groups, which mainly include positively charged groups and hydrophobic groups. Apoorva et al. reported a class of synthetic, peptidomimetic polyurethanes with mimic amino-acid R groups, which could disrupt surface-established biofilms of a broad spectrum of clinically relevant bacteria without being toxic to mammalian cells [13]. In other words, adjusting the hydrophobic properties of the polymer bactericides has proven to be an effective method for enhancing the antibacterial performance against bacteria.

As is known, natural proteins have similar chemical structure to that polymers, which are composed of independent monomer units connecting to each other. Moreover, proteins exhibit much higher biocompatibility in comparison with polymer nanoparticles, because of their non-hemolytic character and good biodegradability. Therefore, replacing the man chain of macromolecules with a bactericidal active protein chain is a promising strategy for the development of novel bactericides. Recently, Akash et al. reported the creation of polymeric nanoparticles with highly efficient antimicrobial properties, demonstrating that the biocompatibility and antibacterial properties of synthesized polymer macromolecules can be regulated by the hydrophobic properties which are highly relevant to the side functional groups and alkyl chain length [14]. Therefore, a potential method appears to be to adjust the chemical structure of chain-like macromolecules to regulate the performance of macromolecule bactericides. Inspired by these studies, it should be a breakthrough step to design a kind of protein bactericide in which both the side groups and the main chain exhibit high antibacterial activity and biocompatibility.

With a wide range of natural sources and low cost, the natural lysozyme is an ideal bactericidal-active biomacromolecular chain with promising application prospects [15,16,17]. However, lysozyme is one kind of alkaline enzyme that hydrolyzes polysaccharides in pathogenic bacteria and is widely present in various tissues and secretions of the human body. It can destroy the main component of bacterial cell wall peptidoglycan, thereby causing bacterial dissolution and death. However, Gram-negative bacterial strains are usually insensitive to the natural lysozyme because they possess a low peptidoglycan content and protection of the outer membrane. Due to the low peptidoglycan content in Gram-negative bacteria, the destructive effect of lysozyme on them is relatively weak. Meanwhile, Gram-negative bacteria have an outer membrane outside their cell wall, and the presence of the outer membrane can prevent antibacterial substances such as lysozyme from entering the interior of bacteria, thereby reducing the bactericidal effect of lysozyme on Gram-negative bacteria. The narrow-spectrum therapy of the natural lysozyme limits its application in clinical medical use. Aiming at these problems, much effort has been devoted to improving the therapeutic effect and antibacterial spectrum of lysozyme, including chemical modification [18], protein fibrillation [19], and controlled drug release [20,21]. For example, Wei et al. investigated the antibacterial activities and mechanism of oligomers and amyloid fibrils formed from hen egg-white lysozyme (HEWL) against *S. aureus* and *E. coli*, while the engineered enzyme activity of modified lysozyme has decreased due to the modification [16]. Kevin J et al. investigated the preparation of cellulose nanocrystal-lysozyme composite films via the method of evaporation-induced self-assembly and broadened the antibacterial spectrum of lysozyme by combining it with other antibacterial active contents [22]. In our previous work, we modified the guanidine group onto the lysozyme chain, improving the positive charge properties to broaden the antibacterial spectrum and enhance antibacterial performance, demonstrating that positive molecule modification was an effective way to improve the antibacterial performance of the natural lysozyme [23]. Herein, we further regulated the hydrophobic properties of modified lysozyme, aiming to enhance the antibacterial activities and broaden the antibacterial spectrum of lysozyme. We designed a hydrophobic modified lysozyme (HML) using a hydrophobic amino acid mimetic monomer library to enhance the antibacterial properties of HML and regulate the alkyl line length of the crosslinking agents to influence the antibacterial activity of HML against Gram-negative bacteria that were insensitive to the natural lysozyme. The HML group was designed to have Leucine-mimetic, Tryptophan-mimetic, and Methionine-mimetic groups as side functional groups to impart hydrophobicity to the modified lysozyme. Accordingly, the HML not only showed an obvious enhancement in antibacterial activity against both Gram-negative and Gram-positive bacteria compared with the natural lysozyme, but also demonstrated less accumulation of bacterial resistance. However, the stability under higher temperature and solution conditions still limited the application of HML.

## 2. Results and Discussion

### 2.1. Construction and Characterization of HML

**Figure 1:**
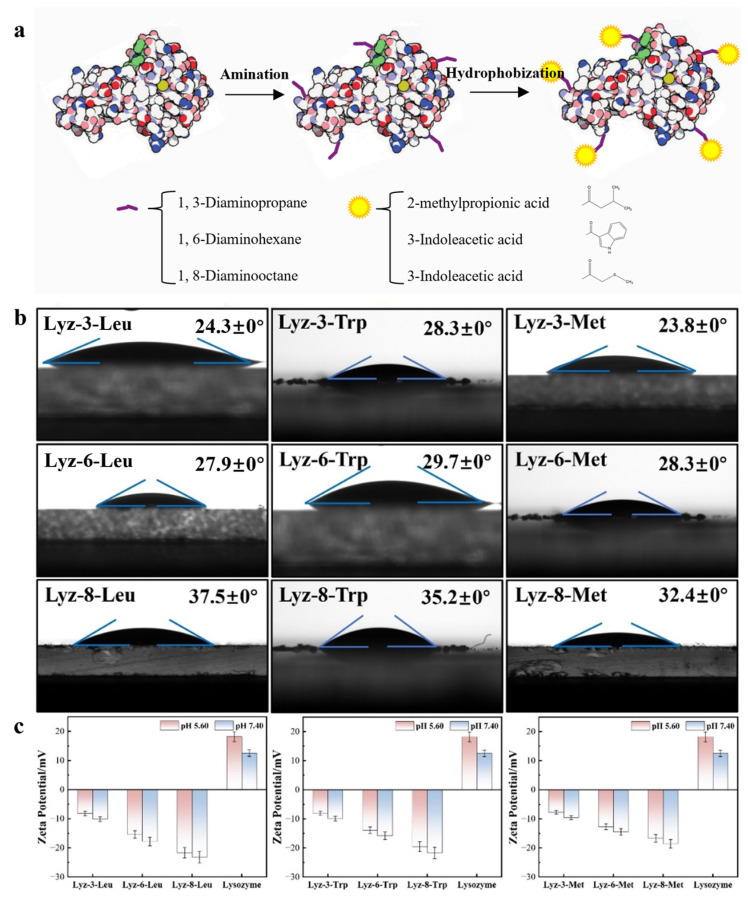
Synthesis process of hydrophobic modified lysozyme (HML) from native lysozyme (**a**). Contact angle measurement of HML Products (**b**). Zeta potential of the designed HML after modification by different amino acids (**c**). Data in c are presented as mean values ± SD, and error bars indicate standard deviations (n = 3 experimental replicates).

**Figure 2:**
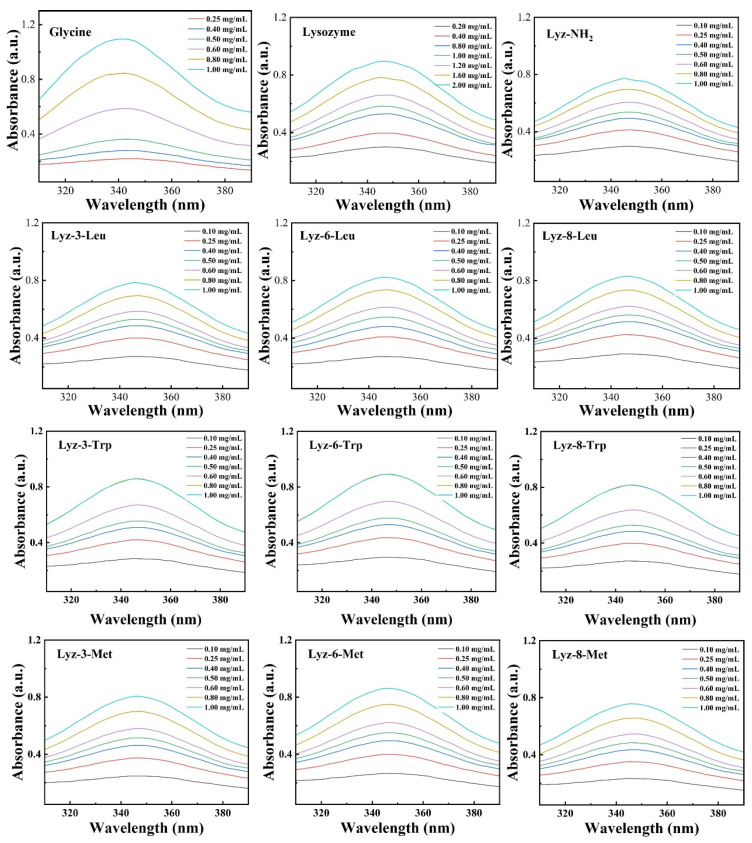
UV-Vis Spectrum of reactive amino groups for glycine, lysozyme, Lyz-NH_2_, and HML.

The precursor of HML bactericides is the natural lysozyme extracted from hen egg whites. As is demonstrated in Figure 1a, the natural lysozyme experienced a process of amination, and the coupling agents used were 1,3-diaminopropane/1,6-diaminohexane/1,8-Diaminooctane with alkyl chains of different lengths of 3, 6, and 8 C-C bonds. The synthesis process showed that diamine substances with alkyl chain lengths more than 10 C-C bonds were hardly dissolved in the reaction solutions and formed two separate phases of oil and water. The product was named as Lyz-x-y, the parameter x represented the number of carbon chain link which was applied as crosslinking agent, and the parameter y represented the mimic hydrophobic functional groups decorated onto the protein chain, including Leu (Leucine), Trp (Tryptophan), and Met (Methionine). Moreover, the modified lysozyme without amination process showed no obvious chemical changes compared to the precursor. In order to statistically analyze the exact number of functional groups modified on the surface of lysozyme, we used 2,4,6-trinitrobenzenesulfonic acid (TNBSA) to perform titration experiments on the number of amino groups linked to the proteins, and the results are shown in Figure 2 and Figure S1 and in Table 1. The D-value between each reaction product and lysozyme represents the number of hydrophobic groups linked with lysozyme, as shown in Table 1. The results demonstrated that the number of functional groups crosslinked with lysozyme decreased when the mass of crosslinking agents increased, mainly because of the increased steric hindrance caused by the extension of the alkyl chains.

In order to explore the hydrophobicity of the HML samples, the surface tension method was applied to test these properties of the HML samples. The samples were dispersed on the surface of the slide several times and dried at room temperature until continuous layers were formed. The test focused on the interface of the sample layer and water to evaluate the hydrophobic properties of the samples. As shown in Figure 1b, after being systematically modified by the functional group, which mimics the R-base groups of the library of hydrophobic amino acids, the hydrophobic properties of the modified lysozyme showed a uniform enhancement in comparison with that of natural lysozyme. In particular, among the hydrophobic properties of the HML samples modified with the same side hydrophobic group, the hydrophobic properties of the modified lysozyme showed an obvious improvement, directly proportional to the length of the alkyl chain, which acts as a coupling agent between the hydrophobic functional groups and the precursor lysozyme. As displayed in Figure S2, DLS has been applied to measure the size of modified lysozymes and found that there appeared to be several particles with a diameter of 500–700 nm, and there also appeared to be signals below 100 nm, which was almost the same as that of the natural lysozyme. We assumed that the solubility had been affected by the modifying function groups and the HML formed large-size particles due to the amphiphilicity of the proteins. Meanwhile, the HML crosslinked with the same length of alkyl chains also presented a slight difference in hydrophobicity owing to the modified groups. As demonstrated in Figure 1c and Figure S3, zeta-potential measurements of the natural lysozyme and HML (16 mg/mL) show that the lysozyme experience an obvious decrease from positive charge of 18.2 ± 1.7 mV for the natural lysozyme to negative charge of −8.2 ± 0.8 mV for Lyz-3-Leu, −15.4 ± 1.3 mV for Lyz-6-Leu, −21.7 ± 1.9 mV for Lyz-8-Leu, −8.1 ± 0.7 mV for Lyz-3-Trp, −13.9 ± 1.1 mV for Lyz-6-Trp, −19.6 ± 1.7 mV for Lyz-8-Trp, −7.7 ± 0.6 mV for Lyz-3-Met, −12.7 ± 1.0 mV for Lyz-6-Met, and −16.7 ± 1.3 mV for Lyz-8-Met at pH 5.60. Meanwhile, the value measured at pH 7.40 went down compared with the above data because of the ionization of lysozyme itself, and it could be concluded that lysozyme retained its own physicochemical properties, which was the basic factor for lysozyme to exhibit its own enzymatic activity properties. These results demonstrated that the hydrophobic groups were successfully modified onto the surface of lysozyme and altered the lysozyme charge from positive to negative as well. Moreover, the extension of the crosslinking alkyl chain also performed the same behavior to alter the surface charge of lysozyme. Furthermore, we applied circular dichroism (CD) to investigate whether the protein structure of lysozyme was changed during the process of chemical modification. The curve shown in Figure S4 demonstrates that the single-α-sheet structure remained stable during the period of chemical modification.

**Figure 3:**
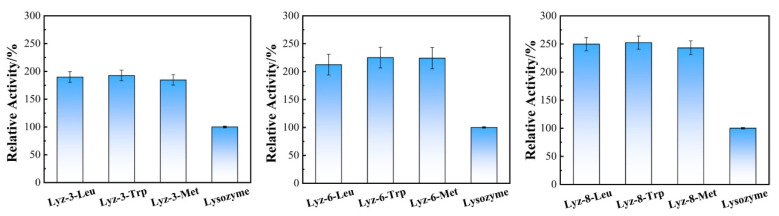
Enzymatic activity of HML bactericide Lyz-3-x, Lyz-6-x, Lyz-8-x, and natural lysozyme. Data are presented as mean values ± SD, and error bars indicate standard deviations (n = 3 experimental replicates).

In order to further test the bactericidal performance of hydrophobic modified lysozyme compared to natural lysozyme precursors, this study conducted enzyme activity measurement experiments on the obtained hydrophobic modified lysozyme samples, and compared them with the one of natural lysozyme. As demonstrated in Figure 3, the enzymatic activity of HML were effectively improved in comparing with natural lysozyme.

### 2.2. Antibacterial Ability of HML Against Gram-Negative Bacteria

**Figure 4:**
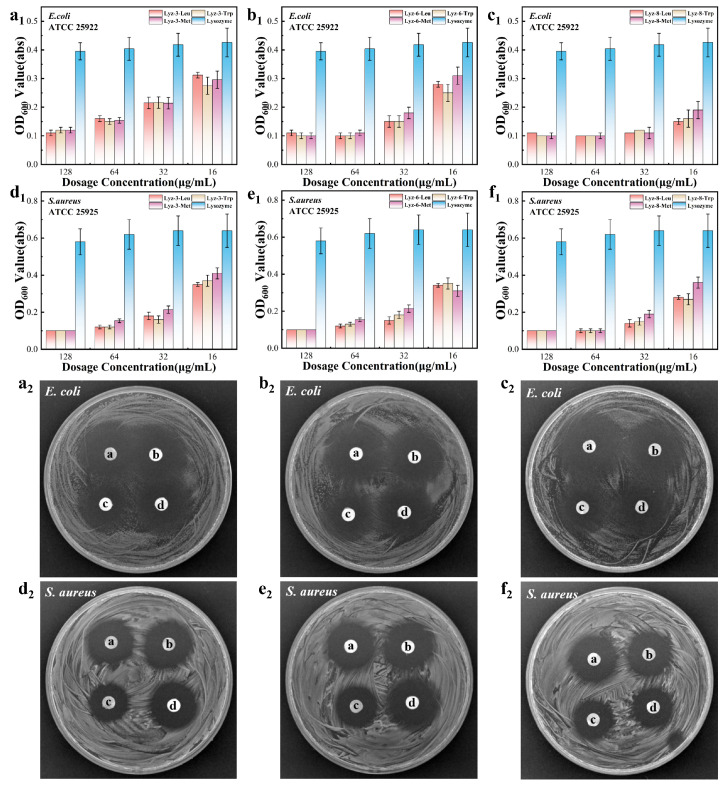
Viability of *E. coli* (ATCC 25922) exposed to Lyz-3-x (**a_1_**), Lyz-6-x (**b_1_**), Lyz-8-x (**c_1_**), and viability of *S. aureus* (ATCC 25925) exposed to Lyz-3-x (**d_1_**), Lyz-6-x (**e_1_**), Lyz-8-x (**f_1_**). The images of bacteriostatic ring test in which *E. coli* (ATCC 25922) were co-cultured at 37 °C for 3 h with Lyz-3-x (**a_2_**), Lyz-6-x (**b_2_**), Lyz-8-x (**c_2_**), and *S. aureus* (ATCC 25925) were co-cultured with Lyz-3-x (**d_2_**), Lyz-6-x (**e_2_**), and Lyz-8-x (**f_2_**), respectively. Data in (**a_1_**,**b_1_**,**c_1_**,**d_1_**,**e_1_**,**f_1_**) are presented as mean values ± SD, and error bars indicate standard deviations (n = 3 experimental replicates).

In order to evaluate the antibacterial activity of the synthesized HML samples against Gram-negative and Gram-positive bacteria, we established a co-culture model experiment. It is known that the natural lysozyme always exhibits low antibacterial activity against Gram-negative bacteria and *S. aureus*, because there exists less β-1,4-glycosidic bond content among Gram-negative bacterial cell walls with which the natural lysozyme effectively reacts. Aiming to solve this problem, we decorated lysozymes with a gradient hydrophobic ability to build a combination with bacterial cell walls by physical effects, enhancing the antibacterial performance against pathogenic bacterial strains. Therefore, *E. coli* (ATCC 25922) was chosen as the Gram-negative bacterial model and *S. aureus* (ATCC 25925) was chosen as the Gram-positive model. These experimental models were co-cultured with folded-up concentrations of the HML samples to evaluate their antibacterial performance. Moreover, Figure 4 demonstrates that the growth of the co-culture experimental models was obviously inhibited in the presence of the HML samples compared to natural lysozymes. In comparison with that of natural lysozymes, HML showed at least a four-fold enhancement in antibacterial activity, which was consistent with the conclusion from the square measure of the antibacterial ring gained by the inhibition zone displayed in Figure 4. The HML with the same hydrophobic groups exhibits an obviously higher antibacterial performance with longer alkyl chains, which also indicates that the product exhibits higher hydrophobicity. Meanwhile, HML with the same alkyl chain length exhibited nearly the same antibacterial performance with varying hydrophobic groups. As a result, the antibacterial properties of HML appear to be more closely related to the length of the crosslinking alkyl chains than to the difference in the modified hydrophobic groups.

In order to quantitatively evaluate the antibacterial efficiency of the reconstructed lysozyme, two parameters are further investigated, namely minimal inhibit concentration (MIC) and minimum bactericidal concentration (MBC). Table 2 demonstrates the results of HML against both Gram-positive and Gram-negative bacteria. Additionally, in comparison with the antibacterial properties of cationic modified lysozyme (CML) synthesized in our previous work [6], we found that the HML possessed higher antibacterial behavior than that of CML. As a result, we confirmed that HML exhibited higher antibacterial performance. Regardless of Gram-positive and Gram-negative bacteria, HML exhibits broad-spectrum antibacterial properties, which makes it a potential bactericide that targets broad-spectrum pathogenic bacteria.

### 2.3. Mechanism of HML Antibacterial Ability

**Figure 5:**
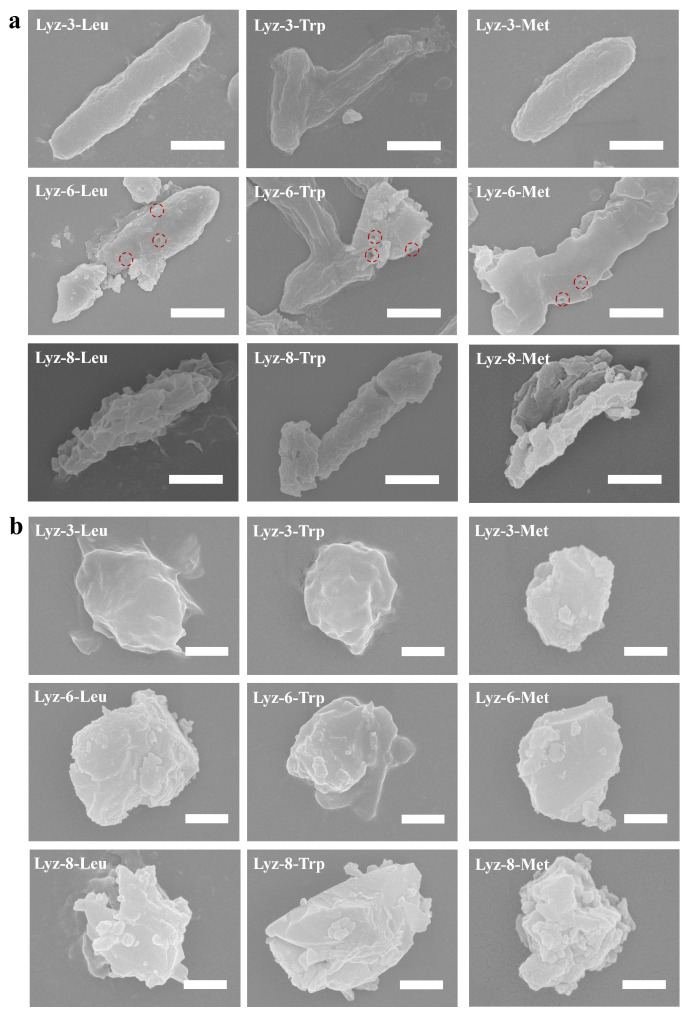
SEM images for *E. coli* (ATCC 25922) treated by HML (**a**), the scale bars of SEM images are 1.0 μm. SEM images for *S. aureus* (ATCC 25925) treated by HML products (**b**), the scale bars of SEM images are 500 nm.

The antibacterial activities of the HML and natural lysozyme were revealed by scanning electron microscopy (Figure 5). For both Gram-negative bacteria *E. coli* and Gram-positive *S. aureus* exposed to 1 mg/mL of HML and Lysozyme, respectively, the morphology of bacterial cell surface can be seen as the antibacterial properties of tested bactericide. Compared with the modified hydrophobic groups, the extension of the crosslinking alkyl chain disrupted the cell walls of both *E. coli* and *S. aureus*. For *E. coli* treated by Lyz-3-x, the surface of the bacterial cell wall remained smooth, but the shape became thinner than the regular ones, and it could be concluded that Lyz-3-x mildly disrupted the structure of the bacterial cells. For *E. coli* treated by Lyz-6-x, a very interesting phenomenon was found: there appeared several obvious pores on the cell-wall of *E. coli*, as indicated by the red rings, and the remnants of bacterial content substances were visible, demonstrating that Lyz-6-x had broken through the bacteria cell wall and caused the leakage of bacterial content substances, representing more effective antibacterial behavior against Gram-negative *E. coli*. For *E. coli* treated by Lyz-8-x, there seemed to be more obvious shrinkage and opening on the bacterial cell wall than in the previous samples, and the shape of the bacteria had already been destroyed, demonstrating that bacterial apoptosis had already occurred. Meanwhile, there was no obvious opening on the cell surface of *E. coli* treated with natural lysozymes (shown in Figure S5) at the same dosage concentration, because the bacterial strains were insensitive to lysozymes. For *S. aureus* exposed to HML, the change in the morphology of coccus followed the same trends as that of *E. coli*, and the cell structure turned to be more broken and fragmented because Gram-positive bacterial cells were sensitive to lysozymes and the coccus had a smaller shape that could sustain less external destruction.

**Figure 6:**
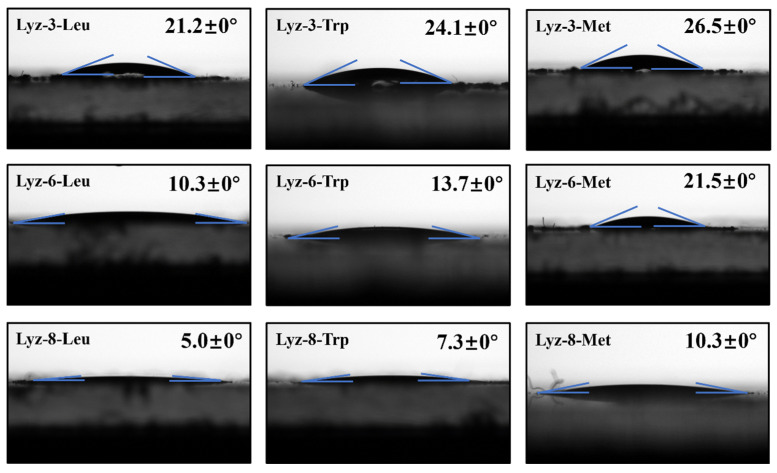
Contact angle measurement of HML samples towards accumulated films of *E. coli*.

It is widely recognized that one of the most effective methods to improve the antibacterial performance of bactericides is to enhance the binding ability between bactericides and bacteria. Similarly, in this study, we attributed the enhanced combining efficiency of the HML to the targeted bacteria by measuring the affinity between HML and bacteria. The surface tension method was applied to test the combing ability of HML towards bacteria. Gram-negative *E. coli* were dispersed on the surface of the slide several times and dried at room temperature until they had formed continuous layers, and this film obviously consisted of the bacteria component. The tests focused on the interface of the bacterial layer, and 1 mg/mL HML solution was used to evaluate the binding ability between HML and *E. coli*. Figure 6 clearly demonstrates that the binding ability between HML and bacteria is obviously higher than that of natural lysozymes (shown in Figure S6), which also means that HML was able to react with the bactericide more effectively. As displayed in Figure 6, the longer alkyl chain modified on the lysozyme led to a higher level of hydrophobicity and assisted the side chains combing to the outer membrane of Gram-negative bacteria owing to hydrophobic interaction, while the HML performed higher antibacterial property in this way.

**Figure 7:**
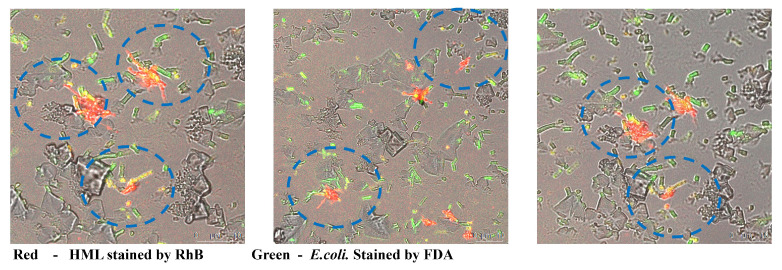
Confocal laser scanning confocal microscopy of stained HML towards stained *E. coli* bacteria.

In order to search the mechanism for improving the antibacterial activity of HML, we applied confocal laser scanning confocal microscopy to monitor the combination between *E. coli* and HML samples. Firstly, we stained the bacteria with Fluorescein disodium (FDA) and gained bacteria exhibiting green fluorescence. Then, we added HML stained by Rhodamine B (RhB) to the bacteria medium. As displayed in Figure 7, we detected the rapid appearance of a yellow fluorescent signal, which represented the superposition of red fluorescence signals and green ones. Meanwhile, there was no similar combining process in the natural lysozyme groups. Moreover, the results above demonstrate that the hydrophobic side chain modification improved the affinity ability between lysozyme and outer cell wall of Gram-negative bacteria.

### 2.4. Therapeutic Selectivity and Hemolysis Activity of HML Bactericide Against Pathogenic Bacteria

**Figure 8:**
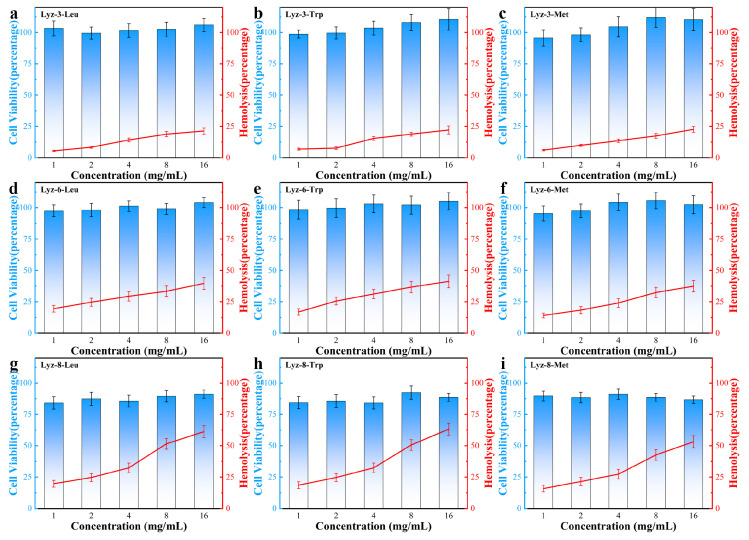
Viability of NIH/3T3 stem cells cultured at 37 °C for 24 h with HML samples and hemolytic activity of HML samples at different concentrations indicates their excellent biocompatibility and non-hemolytic behaviors at relevant therapeutic concentrations. Data in (**a**–**i**) are presented as mean values ± SD, and error bars indicate standard deviations (n = 3 experimental replicates).

After establishing the antibacterial performance of the HML bactericide, we tested cell toxicity in NIH/3T3 mouse-source stem cell lines to determine the biocompatibility of the synthesized HML bactericide. Firstly, the highest tested bactericide concentration was set at 16 mg/mL because the hydrophobic modification obviously affected the solubility in water/saline. The results summarized in Figure 8 showed that HML bactericide had a limited effect on NIH/3T3 stem cells, although the applied concentration (16 mg/mL) has been raised to over 16 times that of its previous MIC, which predicts its potential in medical applications. Lysozyme not only performs the antibacterial-active part in the macromolecule structure, but also provides nutrition for mammalian cells, which was convinced that the cell viability of several groups has increased by over 100%.

Aiming to discuss the hemolysis behavior of HML bactericide, we focused on the red curve in Figure 8 and found that more blood cells broke down while hydrophobicity increased, and HML bactericides with an alkyl chain length over eight C-C bonds all showed hemi-hemolysis, because the hydrophobic side chain was so long that influenced the lipid bilayer structure which is amphiphilic. The difference between cell toxicity and hemolysis is because there exists no obvious organelles in blood cells, which means that they are not able to take lysozyme as nutrition compared with mammalian stem cells. Furthermore, one parameter named therapeutic selectivity (TI = IC_50_/MIC) was calculated to reflect the ability of bactericides to kill pathogenic bacteria, while causing limited toxicity to mammalian cells. The therapeutic selectivity against Gram-negative bacteria are shown in Table 3. The reason why HML bactericide exhibited good biocompatibility and non-hemolysis is that HML bactericide could destroy the polysaccharide components among bacterial cell walls by a natural enzymatic hydrolysis process, while there exists no similar structure in mammalian cells. Therefore, HML could exhibit high antibacterial properties and low biotoxicity at the same time.

**Figure 9:**
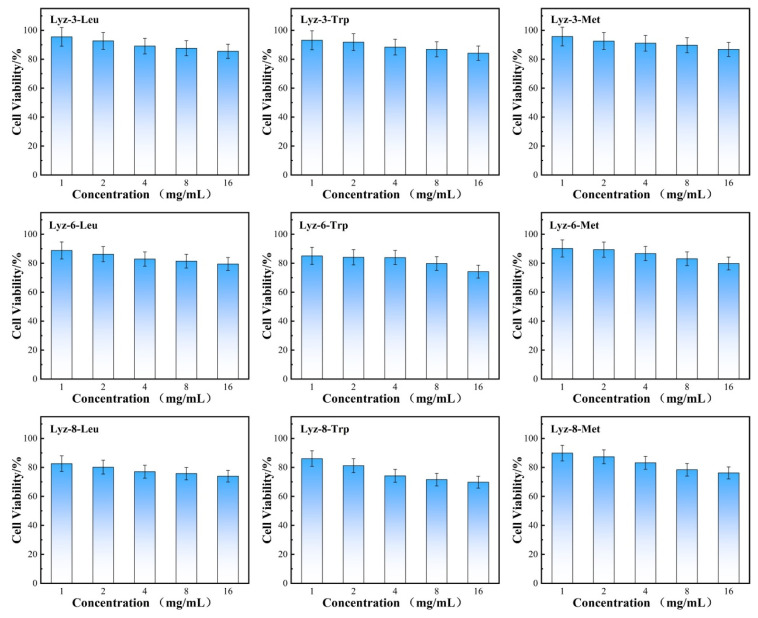
Viability of 293T cells cultured at 37 °C for 24 h with HML different concentrations.

Moreover, aiming to search the biotoxicity and valuate the potential in medical use, we applied 293T cells as human-source experimental subjects of biotoxicity of HML. The results displayed in Figure 9 demonstrate that the viability of human cell under high concentration of HML still remained over 75%. These data confirm that HML has good potential in clinical use.

### 2.5. Bacterial Resistance Development Against Antibiotics Versus HML

**Figure 10:**
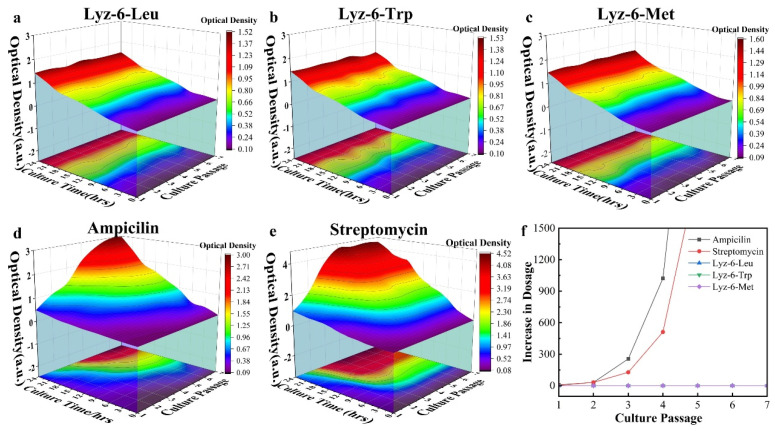
Growth condition of *E. coli* ATCC-25922 treated by sub-MIC of (**a**) Lyz-6-Leu, (**b**) Lyz-6-Trp, (**c**) Lyz-6-Met, (**d**) Ampicillin, and (**e**) streptomycin. (**f**) Resistance development during serial passages in the treatment of sub-MICs (66% of the MIC) of bactericide above, the lines demonstrate the increasing MIC value in the bacteria species harvested at the end of every term of passage.

The abuse of commercial antibiotics and other bactericides in clinical medicine and agriculture use accelerates the process of the natural selection of bacterial species over thousands of times. Bacteria rapidly acquire resistance against commercial antibiotics, limiting their long-term therapeutic effects. Given the membrane disruption mechanism of HML, the development of resistance in bacterial strains would require a complete transformation in the bacterial cell structure, which is obviously impossible. The ability of HML to evade resistance was tested by subjecting pathogenic *E. coli* (ATCC 25922) to multiple serial passages of the sub-MICs (67% of MIC) of three typical HML groups, Lyz-6-Leu, Lyz-6-Trp, and Lyz-6-Met, which were selected based on their antibacterial performance, biocompatibility, and hemolysis. According to Figure 10a–c, the growth curve of bacteria cultured with sub-MICs of Lyz-6-Leu, Lyz-6-Trp, and Lyz-6-Met showed no obvious fluctuation among the whole multiple serial passage periods (ATCC 25922 15 min/generation, 96 generations/day), confirming that bacteria are unable to build resistance against HML. In comparison to ampicillin under the same experimental conditions, Figure 10d,e demonstrate that there was a sharp increase after a few serial passages. All the experiments above led to the conclusion that the bacterial species were far less likely to accumulate resistance towards HML, whereas that of commercial antibiotics showed a rapid decrease. To further evaluate the ability to limit the development of bacterial resistance, the tested bacterial strains were separately harvested, and their corresponding MICs were tested. As shown in Figure 10f, there was no variation in the MICs of bacterial strains treated by HML groups, including Lyz-6-Leu, Lyz-6-Trp, and Lyz-6-Met, during the entire experimental period (~576 generations). The same experiment was carried out using the commercial antibiotic ampicillin. By comparing the results above, there was an over 1500-fold increase in the MIC after only a few passages. It indicates that HML endows an obvious performance increase in limiting the develop of bacteria resistance against bactericide, and guarantees their application value in clinical medicine and agriculture use.

## 3. Materials and Methods

### 3.1. Material

1,3-diaminopropane (>99%, Aladdin, Shanghai, China), 1,6-diaminohexane (>99%, Aladdin), 1,8-diaminooctane (>99%, Aladdin), lysozyme extracted from hen egg-white (Aladdin), N-ethyl-N′-(3-dimethylaminopropyl) carbodiimide hydrochloride (>98%, Aladdin), isovaleric acid (>99%, Aladdin), and 3-indoleacetic acid (>99%, Aladdin) were used as received.

### 3.2. Amination Process of Lysozyme

In order to maintain the stability and alter the surface chemical environment of lysozyme, 10 mM 1,3-diaminopropane/1,6-diaminohexane/1,8-Diaminooctane was firstly dissolved in 10 mL of deionized water to obtain the solution, and the solution was titrated to pH 6.0 by using 3 M HCl. Meanwhile, 10 mL of lysozyme solution (2 mg/mL) was prepared, and was added into the above solution drop by drop. The mixture was then adjusted to pH 6.0, and the coupling process was initiated by adding 100 mg of N-ethyl-N′-(3-dimethylaminopropyl) carbodiimide hydrochloride (EDAC). Another 100 mg of EDAC was added again after 3 h. The pH value was titrated at 6.0. using 3 M HCl, and the reaction was continuously stirred for 6 h. The purification process was achieved by dialyzing the solution with a dialysis bag of 10,000 Mw for 2 days, and the products were collected by freeze-drying and obtained white 17.2 mg dry powder (yield 82%).

### 3.3. Conjugation Process of HML

Aiming to modify the hydrophobic functional groups onto the protein chain of aminated lysozyme, isovaleric acid was set up as the mimic functional group of leucine (Leu), 3-Indoleacetic acid was set up as the mimic functional group of tryptophan (Trp) and 3-Indoleacetic acid was set up as the mimic functional group of methionine (Met). An amount of 10 mM of mimic agents was dissolved in 10 mL of PBS (pH 6.0). Meanwhile, aminated lysozyme (20 mg) was dissolved in 10 mL of PBS (pH 6.0), and was added to the solution above, which made the molecular ratio approximately 100:1. The same initial process was applied to this reaction as described above. The purification process was achieved by dialyzing the mixture with a dialysis bag of 10,000 Mw for 2 days, and the product HML was collected by freeze-drying to obtain 16.1 mg white powder (yield 80.5%).

### 3.4. Function Group Assay of HML

Prepare 500 μL of glycine aqueous solution with the concentrations of 0.25 mg/mL, 0.4 mg/mL, 0.5 mg/mL, 0.6 mg/mL, 0.8 mg/mL, and 1.0 mg/mL, respectively; lysozyme solution and aminated lysozyme with the same relative concentrations; and 500 μL of HML sample solution with concentrations of 0.1 mg/mL, 0.25 mg/mL, 0.4 mg/mL, 0.6 mg/mL, 0.8 mg/mL, and 1.0 mg/mL. Meanwhile, a 5% (*w*/*v*) TNBSA solution was diluted 1000 times using a phosphate-buffered solution (pH 8.0). Next, 1 mL of the diluted TNBSA solution was added to each concentration of glycine samples, lysozyme samples, and HML samples, respectively. After reacting at 37 °C for 2 h, 200 μL of 1 M HCl solution was added to each sample tube. Then, the samples were analyzed using a UV/Vis spectrometer LAMBDA 750 S (PERKINELMER, Wellesley, MA, USA). The absorption and the amount-of-substance concentration of the samples were collected and calculated to obtain a standard concentration curve. Finally, based on the curves obtained above, the amount of hydrophobic groups can be calculated.

### 3.5. Enzyme Activity Assay of HML

Select a single colony of purified micrococcus (CGMCC 1.635) and mix it with 50 mL of LB medium. Incubate the bacteria in a 37 °C constant-temperature shaker at 110 r/min for 18 h. Mix 500 μL of the bacterial solution with 50 mL of liquid LB medium, and shake at 110 r/min for 18 h in a 37 °C shaker to prepare the bacterial suspension. Centrifuge the bacterial suspension (12,000× *g*, 5 min) to collect the bacteria, wash the collected bacteria with PBS buffer (pH 6.2) until no culture medium is present, use a UV spectrophotometer to adjust the instrument wavelength to 450 nm, dilute the washed bacteria with PBS 6.2 buffer to adjust the bacterial concentration, and record the absorbance value at 450 nm.

Accurately weigh 1 mg of solid enzyme and dissolve it in 1 mL of PBS 6.2 buffer, so that the absorbance value changes between 0.025 and 0.125 within 1 min, and the reading should not be less than 1.000 at 1 min. Conduct parallel tests on the same sample for measurement. Add 2.5 mL of bacterial suspension and 0.5 mL of test substance solution to the reaction tube in a constant-temperature water bath at 25 °C. Record the reading A1 at 450 nm at 1 min of reaction, and record the reading A2 at 450 nm at 2 min of reaction. Calculate the corresponding enzyme activity using the following formula:

$$I=\frac{\left|A_{1}-A_{2}\right|}{0.001\times E_{w}}$$

*I*—Enzyme activity;
$\left|A_{1}-A_{2}\right|$—The D-value of absorbance at 450 nm per minute;
*E_w_*—The mass (mg or g) or volume (mL) of the tested enzyme solution contained in 0.5 mL tested solution;
0.001—An activity unit is defined as a decrease of 0.001 in absorbance at 450 nm per minute.

### 3.6. Antibacterial Assay of HML

The minimal inhibitory concentration (MIC) and minimal bactericidal concentration (MBC) were measured to evaluate the antibacterial properties of HML. Samples were dissolved in a series of lysogeny broth (LB) medium tubes at dosages of 2048, 1024, 512, 256, 128, 64, 32, 16, 8, 4, and 2 μg/mL. The original concentration of each group was 2 × 10^5^ CFU·mL^−1^. All experimental groups were composed of 500 μL of serially diluted sample solutions and 500 μL of experimental bacterial suspension. All tubes were shaken at 110 rpm at 37 °C for 24 h (*E. coli*, ATCC 25922, and *S. aureus*, ATCC 25925), and the optical density was used to evaluate the population of col-cultured bacteria in each tube. In addition, 100 μL of bacterial culture medium was collected and plated onto the LB agar plate to examine whether bacterial colonies grew to measure the minimum bactericidal concentration (MBC) of the experimental samples, the plate was photographed by Gel Doc XR+ (Bio-Rad, Hercules, CA, USA).

### 3.7. Scanning Electron Microscopy (SEM)

SEM analysis was performed to observe the effect of the sample on the surface of the bacterial cells using SU8010 (Hitachi, Tokyo, Japan). SEM samples were treated in the steps below: firstly prepare enough 1 × 10^5^ CFU/mL bacterial suspension and bactericide solutions of different samples, then mix them in different experiment groups, and let the mixture stand for 2 min. After centrifugal treatment, white precipitates were collected from each tube, and dispersed with 2.5% glutaraldehyde solution overnight at 4 °C, followed by washing three times with 50 μM PBS (pH 7.4) for 15 min at each step and dehydration with a graded ethanol series (25%, 50%, 75%, 85%, 90%, 100%) for 15 min at each step. The samples were allowed to dry completely at room temperature and then coated with gold NPs (5 nm) by sputtering.

### 3.8. Determination of Viability of NIH-3T3 Stem Cell Co-Cultured with HML

NIH-3T3 mouse-sourced stem cell sample was performed from ProCell, Shanghai. A total of 10,000 NIH-3T3 cells were cultured in Dulbecco’s modified Eagle medium with 10% bovine calf serum and 1% antibiotics at 37 °C in a humidified atmosphere of 5% CO_2_. The cells were then cultured for 24 h. Next, the seeding solutions 10^8^ cells/mL were inoculated into buffered DMEM. The old medium was removed, and 100 µL of the seeding solution was added. The co-cultures were stored in a humidified box with damp paper towels at 37 °C overnight without shaking. HML was diluted in DMEM without antibiotics to obtain the fold-diluted testing concentrations. The old media were replaced with freshly prepared testing solutions with HML and incubated for 24 h at 37 °C. To determine cell viability in the co-cultures, the testing solutions were removed, and the co-cultures were washed with PBS. The samples were stained with Thiazolyl Blue and tested by UV-Vis spectrum to gain the results.

### 3.9. Determination of Hemolysis of HML

We used a previously established protocol to conduct hemolysis assays on Red Blood Cells. Heparin-stabilized rabbit whole blood (pooled, mixed gender) was purchased from BioChannel LLC, NY and processed as soon as received. Next, 10 mL of phosphate-buffered saline (PBS) was added to the blood and centrifuged at 3000× *g* rpm for 5 min. The supernatant was carefully discarded, and red blood cells (RBCs) were distributed in 10 mL of normal saline. This step was repeated at least three times. Purified RBCs were re-distributed in 10 mL of normal saline solution. Next, 0.1 mL of RBC solution was added to 0.4 mL of HML solution in NS in a 2 mL tube and mixed by shaking gently. RBCs incubated with NS and water were used as negative and positive controls, respectively. The mixture was incubated at 37 °C for 1 h, with shaking at 120 rpm. After the incubation period, the solution was centrifuged at 3000× *g* rpm for 5 min, and 100 µL of the supernatant was transferred to a 96-well plate. The absorbance of the supernatant was measured at 570 nm using UV–Vis spectroscopy.

### 3.10. Determination of Bacterial Resistance Development

The bacterial strains of *E. coli* (ATCC 25922) were chosen as the experimental model and inoculated in LB medium with 2/3 of the MIC measured in previous experiments at 37 °C and 220 rpm for 24 h (~96 bacterial generations for one serial passage), and were monitored to obtain the bacterial growth curve. The bacterial strains were then separately harvested and tested for MIC using the same method as above to determine the MIC of the HML sample.

## 4. Conclusions

In summary, we reported a method to generate a series of hydrophobically modified lysozymes, which were synthesized by adjusting the hydrophobic functional groups on the surface of lysozyme. Thus, the binding capacity of HML and Gram-negative *E. coli* has been proven to increase and assist in improving the antibacterial performance of HML. We confirmed that the synthesized bactericides effectively killed Gram-negative bacteria, which could not be achieved by natural lysozyme, and improved the antibacterial effect over four times compared with that of natural lysozyme. HML was also confirmed to have low hemolysis and low toxicity in order to explore to mammalian cells, and the therapeutic index was over 500, confirming its potential in medical applications. Most importantly, bacteria were far less likely to accumulate resistance towards synthesized HML, which makes it more advantageous than antibiotics. Therefore, it is expected that the therapeutic protein-based bactericide we present could be a highly promising antibacterial agent with potential applications in food safety, agriculture, and clinical medicine.

## Figures and Tables

**Table 1:** Determination of reactive amino groups amount for glycine, lysozyme, Lyz-NH_2_, and HML.

Sample	Conc._A340=0.40_	Conc._A340=0.40_	Conc._A340=0.40_	Amount_amino_ (C_Gly_/C_x_)	Amount_Hydrophobic_
Glycine	0.610	0.835	1.06	-	-
Lysozyme	0.0313	0.0523	0.0733	16.64	-
Lyz-3-Leu	0.0162	0.0292	0.0422	30.45	13.81
Lyz-3-Trp	0.0160	0.0263	0.0366	32.94	16.30
Lyz-3-Met	0.0210	0.0319	0.0428	26.66	9.96
Lyz-6-Leu	0.0178	0.0288	0.0398	29.96	13.32
Lyz-6-Trp	0.0143	0.0244	0.0345	35.87	19.23
Lyz-6-Met	0.0210	0.0263	0.0366	29.92	13.28
Lyz-8-Leu	0.0184	0.0293	0.0401	30.20	13.56
Lyz-8-Trp	0.0184	0.0292	0.0401	29.39	12.75
Lyz-8-Met	0.0237	0.0354	0.0471	23.94	7.30

**Table 2:** Minimum inhibitory concentration (MIC) and minimum bactericidal concentration (MBC) values of HML.

Sample	MIC*_E.__coli_* (μg/mL)	MBC*_E.__coli_* (μg/mL)	MIC*_S__. aureus_* (μg/mL)	MBC*_S__. aureus_* (μg/mL)
Lyz-3-Leu	128	128	128	256
Lyz-3-Trp	128	128	128	256
Lyz-3-Met	128	256	128	256
Lyz-6-Leu	64	128	128	128
Lyz-6-Trp	64	128	128	128
Lyz-6-Met	128	128	128	256
Lyz-8-Leu	32	64	64	64
Lyz-8-Trp	32	64	64	64
Lyz-8-Met	64	64	64	64
Lyz-3-Gua	256	512	256	512
Lyz-6-Gua	128	256	128	256
Lyz-8-Gua	128	128	128	256

**Table 3:** Therapeutic index (TI, TI = IC_50_/MIC) of HML against *E. coli* (ATCC 25922) and *S. aureus* (ATCC 25925).

Sample	TI*_E. coli_*	Ti*_S. aureus_*	Sample	TI*_E. coli_*	Ti*_S. aureus_*	Sample	TI*_E. coli_*	Ti*_S. aureus_*
Lyz-3-Leu	>500	>500	Lyz-6-Leu	>500	>500	Lyz-8-Leu	250	250
Lyz-3-Trp	>500	>500	Lyz-6-Trp	>500	>500	Lyz-8-Trp	500	500
Lyz-3-Met	>500	>500	Lyz-6-Met	>500	>500	Lyz-8-Met	>500	>500

## Data Availability

Data are contained within the article and Supplementary Materials.

## Supplementary Materials

The following supporting information can be downloaded at: https://www.mdpi.com/article/10.3390/molecules30020232/s1, Figure S1. Determination of reactive amino groups amount for Glycine, Lysozyme, Lyz-NH_2_ and HML; Figure S2. Size distribution of the designed HML after modifying by different amino acids; Figure S3. Zeta potential distribution of the designed HML after modifying by different amino acids; Figure S4. Circular Dichroism Curve of Lyz-6-Trp, Lyz-6-Leu, Lyz-6-Met; Figure S5. SEM images of *E. coli* treated by natural lysozyme; Figure S6. Contact angle measurement of natural lysozyme towards accumulated films of *E. coli*.
