# Supplementary material for: Enhanced Antibacterial Activity of Hydrophobic Modified Lysozyme Against Gram-Negative Bacteria Without Accumulated Resistance
Source: Molecules. 2025 Jan 9;30(2):232. doi: 10.3390/molecules30020232 (PMC11767388; doi:10.3390/molecules30020232)
Supplement: Supplementary file 1 [file molecules-30-00232-s001.zip › molecules-3403649-supplementary.pdf]

Enhanced Antibacterial Activity of Hydrophobic Modified Lysozyme Against Gram-Negative Bacteria Without Accumulated Resistance

Zhenhui Li, Song Lin, Mei Zhu, Xiaoman Liu and Xin Huang *

School of Chemistry and Chemical Engineering, Harbin Institute of Technology, Harbin 150001, China; lizhenhui1993@163.com (Z.L.); 18204623951@163.com (S.L.); zhumeipolymer@163.com (M.Z.); liuxiaoman@hit.edu.cn (X.L.)

* Correspondence: xinhuang@hit.edu.cn

Supporting Information

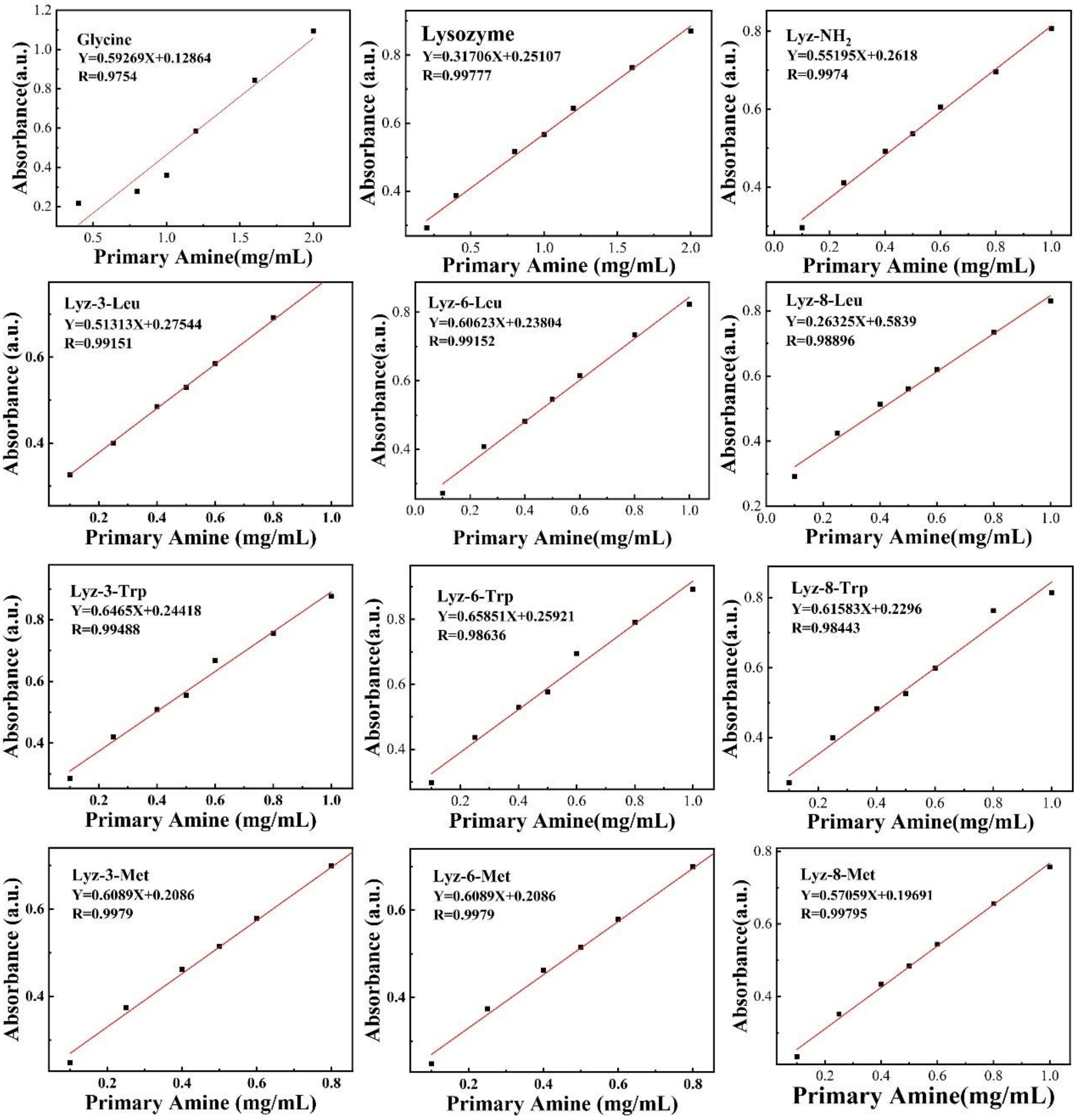

Figure S1. Determination of reactive amino groups amount for Glycine, Lysozyme, Lyz-NH_2 and HML.

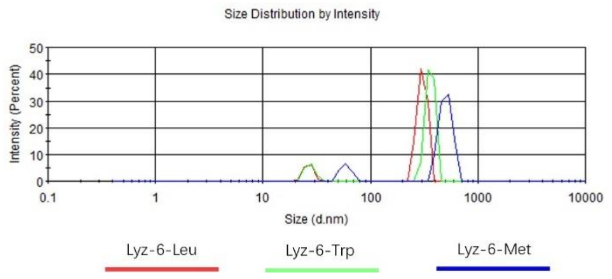

Figure S2. Size distribution of the designed HML after modifying by different amino acids.

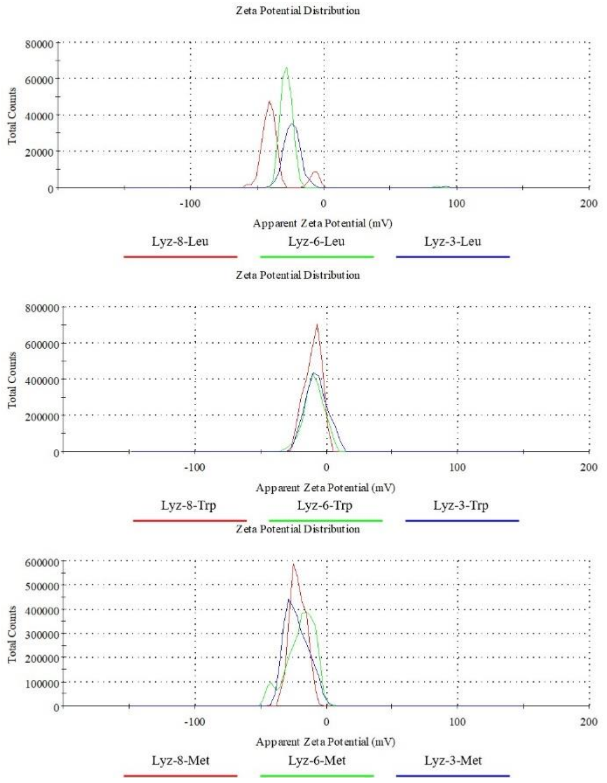

Figure S3. Zeta potential distribution of the designed HML after modifying by different amino acids

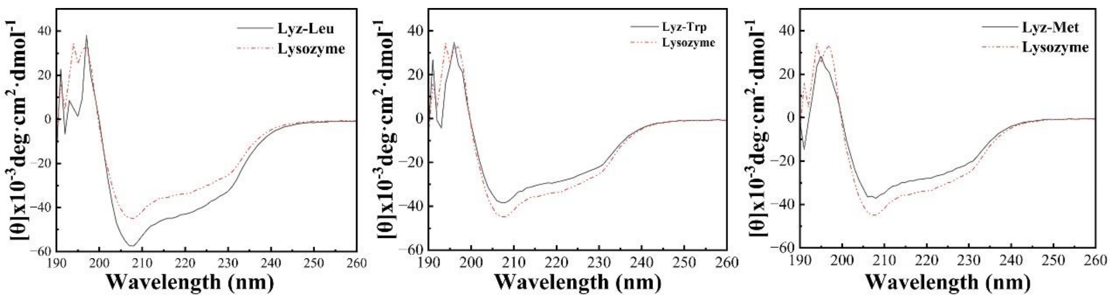

Figure S4. Circular Dichroism Curve of Lyz-6-Trp, Lyz-6-Leu, Lyz-6-Met.

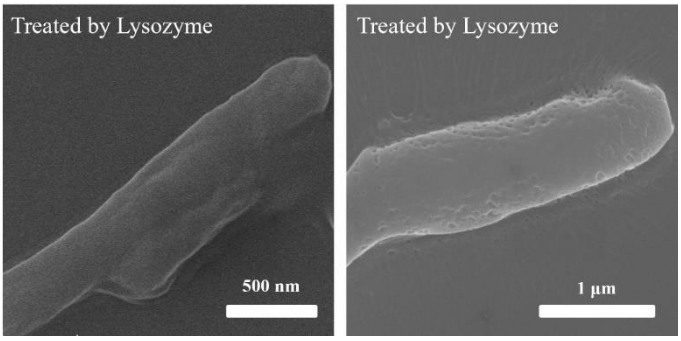

Figure S5. SEM images of *E. coli* treated by natural lysozyme.

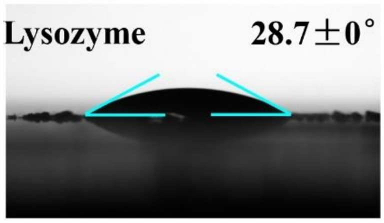

Figure S6. Contact angle measurement of natural lysozyme towards accumulated films of *E. coli*.
